# Conducting Virtual, Synchronous Focus Groups Among Black Sexual Minority Men: Qualitative Study

**DOI:** 10.2196/22980

**Published:** 2021-02-08

**Authors:** Derek T Dangerfield II, Charleen Wylie, Janeane N Anderson

**Affiliations:** 1 Johns Hopkins School of Nursing Baltimore, MD United States; 2 Us Helping Us, People Into Living, Inc Washington DC, MD United States; 3 Department of Health Promotion and Disease Prevention College of Nursing University of Tennessee Health Science Center Memphis, TN United States

**Keywords:** engagement, recruitment, sexual health, telehealth

## Abstract

**Background:**

Focus groups are useful to support HIV prevention research among US subpopulations, such as Black gay, Black bisexual, and other Black sexual minority men (BSMM). Virtual synchronous focus groups provide an electronic means to obtain qualitative data and are convenient to implement; however, the protocols and acceptability for conducting virtual synchronous focus groups in HIV prevention research among BSMM are lacking.

**Objective:**

This paper describes the protocols and acceptability of conducting virtual synchronous focus groups in HIV prevention research among BSMM

**Methods:**

Data for this study came from 8 virtual synchronous focus groups examined in 2 studies of HIV-negative BSMM in US cities, stratified by age (N=39): 2 groups of BSMM ages 18-24 years, 5 groups of BSMM ages 25-34 years, and 1 group of BSMM 35 years and older. Virtual synchronous focus groups were conducted via Zoom, and participants were asked to complete an electronic satisfaction survey distributed to their email via Qualtrics.

**Results:**

The age of participants ranged from 18 to 44 years (mean 28.3, SD 6.0). All participants “strongly agreed” or “agreed” that they were satisfied participating in an online focus group. Only 17% (5/30) preferred providing written informed consent versus oral consent. Regarding privacy, most (30/30,100%) reported “strongly agree” or “agree” that their information was safe to share with other participants in the group. Additionally, 97% (29/30) reported being satisfied with the incentive.

**Conclusions:**

Conducting virtual synchronous focus groups in HIV prevention research among BSMM is feasible. However, thorough oral informed consent with multiple opportunities for questions, culturally relevant facilitation procedures, and appropriate incentives are needed for optimal focus group participation.

## Introduction

Disparities in HIV and negative sexual health outcomes have impacted Black gay, Black bisexual, and other Black sexual minority men (BSMM) throughout the United States for more than a decade [1,2]. From 2014 to 2018, the incidence of HIV infections have remained unchanged among BSMM generally and have increased by 12% for BSMM ages 25-34 years [3]. In 2018, BSMM accounted for 37% of new diagnoses among gay and bisexual men [3]. If infection rates remain stable and treatment and prevention activities remain ineffective, estimates suggest a 50% lifetime risk of infection among BSMM [4]. More culturally relevant, high-impact activities to reduce HIV and improve health outcomes for this group are urgently needed [5]. The impact of prevention strategies such as pre-exposure prophylaxis (PrEP) for HIV-negative men and antiretroviral therapy for men living with HIV could be optimized if BSMM are actively engaged in designing intervention approaches [6-8]. Focus groups can be a particularly useful method for identifying community needs and designing culturally relevant intervention strategies [8-10].

Focus groups use within-group perspectives, discussions, and interactions to gather information about collective views on a topic [8,10]. Individual in-depth interviews gather detailed, personal information regarding topics that are sensitive or about which little is known [11], while focus groups leverage group dynamics to identify community views and design culturally relevant interventions [8,11]. Studies using focus groups have uncovered nuanced sociocultural experiences, such as how the combined impact of racism, homonegativity, and stigma from society, family, and health care providers create barriers to HIV prevention behaviors among BSMM [8,10,12]. Focus group data have also revealed how psychosocial factors, such as medical mistrust, inhibit participation in prevention activities, HIV treatment, and research among BSMM [8,12,13].

Although using in-person focus groups to design interventions for BSMM is popular [10], social distancing and stay-at-home orders due to COVID-19 have prohibited in-person research protocols, which could affect progress toward obtaining the high-quality data needed to design culturally relevant strategies for BSMM. Remote research protocols are needed to continue HIV prevention and health promotion activities. Virtual focus groups (ie, focus groups that are not in person) provide an electronic means to obtaining qualitative data from several participants simultaneously [14,15].

Virtual focus groups provide an online platform by which participants can respond to a series of open-ended questions similar to an in-person focus group [16]. Virtual focus groups refer to online chatrooms, discussion boards, email correspondence [14,15,17], and more recent computer-mediated, video communication modalities such as Skype (Microsoft) and Zoom (Zoom Video Communications) [16,18,19]. Conducting virtual focus groups could help researchers overcome various challenges related to study location, research costs, and obtaining the views from a range of participants within a population [20,21]. Virtual focus groups can also reduce inhibitions among some participants and allow more free-flowing discussions [20]. Some have found that the anonymous setting of virtual focus groups allows some participants to discuss sensitive topics more candidly than they would do in person [16,21]. Recent studies have briefly described the protocols and outcomes for conducting virtual focus groups for some subpopulations, such as youths [22], transgender men and women [16,23], and gay and bisexual men [21]. White and colleagues [8] have also briefly described in-person focus group research conducted with BSMM in HIV prevention research. However, little is known regarding the protocols for conducting virtual synchronous focus groups or the acceptability of conducting this research among BSMM.

Describing the protocols and exploring the feasibility and acceptability of conducting virtual synchronous focus group research among BSMM is crucial to obtaining quality data and conducting ethical research [8,24]. It is well established that medical and research mistrust persists among BSMM [8,12,13] which could limit optimal research using virtual synchronous focus groups for this population. The intersectional experience of being both a sexual and racial minority in the United States also inhibits optimal research participation among BSMM [8,25]. Obtaining optimal research participation from BSMM requires careful consideration, as few researchers have had substantial success in reaching or engaging BSMM in HIV research [6,8,26,27]. Although some have described the general use of Zoom videoconferencing for qualitative data collection [16,18,19,28], the literature describing ethical research protocols using virtual synchronous focus groups among BSMM in HIV prevention research is lacking.

To address this deficit, this paper describes the protocols and acceptability of conducting virtual synchronous focus groups in HIV prevention research among BSMM. To date, the methodological and ethical issues related to recruitment and screening, informed consent, maintaining privacy, focus group facilitation, and providing incentives for conducting virtual synchronous focus groups with BSMM have not been adequately addressed. The procedures and protocols in conducting virtual synchronous focus group studies require clarification because research designs, teams, and protocols impact study participation, group dynamics, and the quality of responses among BSMM in HIV prevention research [8,10]. Details from this study will improve researchers’ ability to ethically gather in-depth, culturally relevant, and high-quality data to design interventions to reduce HIV for BSMM.

## Methods

### Virtual Focus Group Sample

Data for this study came from 8 virtual synchronous focus groups examined in 2 studies of HIV-negative BSMM in US cities conducted from March 2020 to June 2020 (N=39). One study explored BSMM’s preferences for a multicomponent, peer-based intervention to increase HIV-risk perceptions and PrEP initiation; the other study explored ethical and culturally responsive modalities to improve PrEP research participation among BSMM. Eligibility for both studies included being at least 18 years of age (the age limit for one study was 35 years), identifying as Black or African American and as a man, reporting having sex with a male partner in the previous 12 months, self-reporting being HIV-negative, and residing in a US city. Both studies were guided by the life course theoretical framework. Groups were stratified by age, resulting in the following age groups: 18-24 years (2 groups), 25-34 years (5 groups), and 35 years and older (1 group). Each focus group lasted between 60 and 90 minutes and was conducted online via Zoom. At the end of the focus group, participants were asked to complete an electronic satisfaction survey distributed to their email through a private Qualtrics link. The survey items were designed with consideration of the domains of focus group research conduct and asked questions related to participant satisfaction with the online modality, comfort, concerns regarding privacy, and study incentives. All study procedures were approved by the Johns Hopkins School of Medicine Institutional Review Board. Virtual synchronous focus group procedures were conducted in the same manner for both studies and are described in the following section.

### Recruitment and Screening

Participants were recruited from a combination of active and passive recruitment strategies. Passive recruitment included sharing institutional review board (IRB)–approved study fliers and study information on social media websites (eg, Facebook, Twitter) and Craigslist. Active recruitment included reaching out to participants from existing studies, contacting local community-based organizations, and using incentivized referrals from participants within the study. Interested volunteers could contact the study via phone or text messages and were provided additional study-related information by a trained research assistant using an IRB-approved screening script. The same research assistants screened participants for both studies. After questions were answered, research assistants screened volunteers for eligibility and provided eligible volunteers the details related to the time and log-in details for the virtual synchronous focus group.

### Informed Consent

After eligibility was determined, research assistants emailed eligible volunteers a copy of the IRB-approved oral consent form detailing the nature of the study, explaining the potential risks involved in study participation, and indicating that their participation was voluntary. Volunteers were provided an opportunity to ask questions about the study and protocol to the research assistants and the principal investigator (PI) prior to, during, and after the study. After all scheduled study participants joined the virtual focus group, the PI reviewed study-related activities as described in the oral consent form and gave everyone the opportunity to ask additional questions verbally or within the private chat function in Zoom. The PI then prompted each individual participant to provide verbal informed consent. Once the audio recording began, all participants were again asked to individually confirm that they were providing verbal informed consent detailing the nature and risks of the study, that their participation was voluntary, and that they could discontinue at any time without consequence. The study team documented the verbal informed consent for each participant in writing.

### Establishing and Maintaining Privacy

Once we determined their eligibility, participants were emailed a copy of the oral informed consent document and instructions informing them of the virtual format, that they should be in a private location, and to use headphones to protect their and others’ information. They were also notified that they might be asked to show the group their location via the reverse camera function on their mobile phone or internet-connected device (eg, computer, tablet) to confirm that they were alone. The research team (ie, the research assistants and the PI) informed participants that if their location was compromised by another individual entering their space they would be removed from the virtual focus group.

Participants were provided a password-protected Zoom link and instructed not to share the link with anyone. Once all participants joined the virtual focus group, the facilitator asked each person to verbally confirm that he was in a private location to share and receive information. Participants (including facilitators) were asked to confirm their privacy by showing everyone their space on camera. This was not done if participants were alone in their cars, had headphones on, or were noted walking around their space with their phone or tablet prior to the focus group meeting with no one visible in the background. All participants were also asked if they believed the study environment (eg, facilitators, other participants) to be safe enough to share their views.

Once the facilitator and participants confirmed the group’s safety and privacy, the facilitator locked the meeting using Zoom’s “lock meeting” function and informed the participants that the meeting was locked. Participants were then asked to change their Zoom screen name to a pseudonym (eg, name of their favorite color) to limit the risk of a participant’s given name being audio recorded or exposed in a computer screen shot.

### Virtual Focus Group Facilitation

Semistructured focus group guides were designed in consultation with key informants, local community-based organization leadership and staff members who had strong ties to the target population, and investigators with expertise in HIV prevention, qualitative research, and health communications. Participants who accessed a virtual synchronous focus group using an internet-connected mobile device (eg, cellular phone, tablet) were instructed to charge their mobile device while in the focus group to maintain connection to the meeting. Given the relative newness of virtual synchronous focus groups as a data collection modality among this population, focus groups in both studies were limited to 5 participants to reduce the risk of potential privacy breaches and to increase the ease of facilitation. Focus groups were recorded using a digital audio recorder placed near the facilitators’ computer to ease participants’ concerns of being video recorded.

Two experienced facilitators conducted the virtual groups. One facilitator, a self-identified BSMM with experience conducting qualitative research among the population, led the discussion, managed the group, and recorded field notes. The other facilitator scheduled the groups, recorded field notes, observed group dynamics, and provided technical support for participants who had difficulties connecting to the meeting (eg, mistaken password, confirming time and attendance). Focus groups were also conducted on weekends to accommodate participant schedules. Prior to the discussion, the facilitator initiated casual conversations with the participants virtually to increase their comfort and build rapport prior to the formal discussion. Participants were provided an opportunity to ask additional questions about the study or procedures and to debrief with the facilitators regarding their attitudes toward the nature of the study and the online modality before and after the meeting, which was documented in field notes. Each group began with the facilitator discussing the purpose and ground rules for discussion (eg, one person speaks at a time, respect each other's comments, maintain privacy). For both studies, participants were asked targeted questions related to ethical research conduct and how researchers could better engage with BSMM during focus groups.

### Incentives

Participants were compensated with a US $80 electronic Amazon gift card for one study and a US $75 electronic Amazon gift card for another study. For both studies, participants who referred other eligible volunteers were compensated an additional US $40 electronic gift card for each eligible volunteer they referred up to 2 referrals. Gift cards were scheduled for dissemination within 14 business days of focus group participation and delivered directly to the participants’ email on file.

### Analysis of Research Protocols and Participant Satisfaction

Field notes that were documented by the research team were reviewed and organized through a process of abductive analysis, and the notes were closely analyzed with consideration to relevant frameworks for qualitative and HIV prevention research methodology [8,29,30]. Specifically, notes from each group were independently reviewed by the authorship team, and then themes related to the procedures and pragmatic issues of the groups were discussed by the research team. Age-related differences were also considered during analysis. Themes were identified through reflexive debriefing whereby the research team outlined and agreed upon pertinent, salient domains for virtual focus group conduct [8,31]. Themes were also considered relative to participant responses to the satisfaction survey.

## Results

Table 1 reports the responses from the satisfaction survey regarding virtual synchronous focus group participation among BSMM. Of the 39 who participated in the groups, 30 (77%) completed the survey. The age of participants ranged from 18 to 44 years (mean 28.3, SD 6.0). Regarding virtual focus group participation, 86% (26/30) reported that they “strongly agreed” that they were satisfied participating in a focus group online, while the remaining 14% (4/30) reported “agree.” Most (23/30, 77%) reported not preferring in-person focus group participation.

**Table 1:** Satisfaction of virtual synchronous focus group participation among Black sexual minority men (N=30).

Participants' age and responses		Value
Age (years), range		18-44
Age (years), mean (SD)		28.3 (6.0)
**I was satisfied with participating in a focus group online, n (%)**		
	Strongly agree	26 (87)
	Agree	4 (13)
**I understood the purpose of the study, n (%)**		
	Strongly agree	26 (87)
	Agree	4 (13)
**I would have preferred to provide written informed consent than provide verbal informed consent, n (%)**		
	Strongly agree	3 (10.0)
	Agree	2 (7)
	Neither agree nor disagree	11 (37)
	Disagree	4 (13)
	Strongly disagree	10 (33)
**I would have preferred to participate in the focus group in person, n (%)**		
	Strongly agree	3 (10)
	Agree	4 (13)
	Neither agree nor disagree	11 (37)
	Disagree	6 (20)
	Strongly disagree	6 (20.0)
**In the future, I would like to participate in other online focus groups, n (%)**		
	Strongly agree	19 (63)
	Agree	10 (33)
	Neither agree nor disagree	1 (3)
**It is more feasible for me to participate in an online focus group than in an in-person focus group^a^, n (%)**		
	Strongly agree	13 (45)
	Agree	9 (31)
	Neither agree nor disagree	4 (14)
	Disagree	3 (10)
**I felt that my information was safe to share with other participants in the group, n (%)**		
	Strongly agree	20 (67)
	Agree	10 (33)
**I believe my information will be kept confidential by the research team, n (%)**		
	Strongly agree	19 (63)
	Agree	10 (33)
	Neither agree nor disagree	1 (3)
**I believe the other participants were in a private space, n (%)**		
	Strongly agree	18 (60)
	Agree	11 (37)
	Neither agree nor disagree	1 (3)
**I believe my information will be kept confidential by the other people who participated in the focus group, n (%)**		
	Strongly agree	17 (57)
	Agree	10 (33)
	Neither agree nor disagree	3 (10)
**I was satisfied with the incentive I received for participating in the study^a^, n (%)**		
	Strongly agree	13 (43)
	Agree	15 (50)
	Disagree	1 (3)

^a^Due to missing data, some responses are less than the total sample.

The following section describes the themes from the research team’s debriefing of participant responses.

### Motivations and Barriers to Focus Group Participation

Most participants were between ages 25 and 34 years. During screening, research assistants noted that younger participants lacked private locations to participate in a sexual health focus group focused on BSMM. Approximately one-third of participants explicitly mentioned interest in study participation because members of the investigative team were Black and the PI was a BSMM. For instance, one participant, age 30 years, said, “That’s why I wanted to do this, because you will understand what we’re saying better than ‘them’ and you need this information.” During focus groups, several others reported having expectations that the research team would better understand their perspectives and needs as BSMM, and they were comfortable sharing more personal information with the research team members than they typically would do in research studies with predominately White investigators. In several focus groups, participants expressed feeling as if non-Black researchers “don’t really care about us.”

### Informed Consent

Of those surveyed, 33% (10/30) reported “strongly disagree” to a preference of providing written informed consent versus the oral consent they provided; 13% (4/30) reported “disagree,” and 37% (11/30) reported “neither agree nor disagree.” Field notes documented how participants across age groups asked questions only related to the nature of the audio recording (whether their faces would be recorded via Zoom) and how long after their participation they would receive their incentive.

### Establishing and Maintaining Privacy

All participants reported that they “strongly agree” or “agree” that their information was safe to share with other participants in the group; 63% (19/30) “strongly agreed” that they believed their information would be kept confidential by the research team; 33% (10/30) reported “agree.” Most (18/30, 60%) strongly agreed that they believed other participants were in a private space while participating in the group, and 37% (11/30) “agreed.” Of note, to maintain privacy, 2 participants between ages 25 and 34 years participated in the groups in their cars. Only 1 person (from the 25-34 year age group) was removed from the virtual focus group due to a combination of technical difficulties and a suspicious location that prompted the other participants to express concerns privately in the chat box.

### Virtual Focus Group Facilitation

The team noted that the domains of the focus group guide were maintained despite the virtual nature of the research in both studies. The facilitator had to remind participants across age groups to speak up to ensure that the audio recorder could capture the conversation. Since background noises distracted the audio recording and since excited participants would occasionally speak over each other, participants were also reminded to speak one at a time and to mute themselves if they were not speaking.

The study team had no record or impression that the virtual modality limited participants’ sharing their views. Across age groups, participants adequately responded to focus group questions and referred to each other by the pseudonym in the Zoom chat, respecting each other’s privacy for the audio recording. Postinterview debriefing with participants revealed that groups with men 34 years old and under indicated increased comfort in participating in the focus group due to the small size (5 participants or less), although participants mentioned they would also prefer a small size in person.

### Incentives

Among the participants, 45% (14/30) reported strongly agreeing that they were satisfied with the incentive, and 52% (16/30) reported “agree.” However, due to administrative barriers, some groups did not receive their incentive for approximately 30 days after their participation, which caused participants to continue to reach out to the investigative team and make formal complaints. Two participants reached out to the IRB with concerns that the research team was taking advantage of their participation and did not believe the investigative team would compensate them for their time. This caused the research team to identify ways to immediately compensate participants after their virtual research conduct.

## Discussion

This paper outlines the protocols for conducting virtual synchronous focus groups with BSMM for HIV prevention research and provides quantitative and qualitative feedback on acceptability from participants. Overall, conducting virtual synchronous focus groups in HIV prevention research among BSMM is feasible. However, careful consideration and attention to providing informed consent, ensuring privacy, facilitating groups, and promptly providing incentives is necessary for optimal focus group participation. Focus group facilitators must be explicitly trained to thoroughly explain study goals and research protocols, ensure privacy, and manage virtual synchronous groups with BSMM.

Conducting virtual synchronous focus groups could be a useful modality to recruit and engage BSMM who are otherwise “hard to reach,” such as professional men who are unable to attend research offices during the workday and low-resourced men who may not have transportation to travel to research facilities [32]. As many studies of BSMM in HIV prevention research oversample low-resourced participants, virtual synchronous focus groups could facilitate data collection among a more representative sample. However, we did not document participants’ socioeconomic status in this study and were not able to quantify the relative yield of recruitment for virtual research participation compared to in-person activities; others have found no substantial difference in recruitment yield for virtual versus in-person activities [19]. Moreover, COVID-19 impacted study recruitment, as clinics that were typically helpful were only accepting scheduled patients for in-person visits (not research staff) and staff members that could have referred patients to the studies were limited.

Providing IRB-approved oral informed consent was feasible and may be more acceptable among BSMM than obtaining written informed consent for virtual, synchronous focus group participation. This could be due to the convenience of not having to provide electronic or written signatures. Acceptability could also be due to the detailed nature of our informed consent process that provided several opportunities for study participants to ask questions and gain clarification about study-related expectations. However, it is important that the research team uniformly document oral informed consents. Although most participants reported being satisfied with the informed consent process, more information is still needed about the differences, if any, in BSMM’s comprehension of study protocols when informed consent is obtained orally versus in written form. Providing multiple opportunities for participants to ask questions could build trust in the research and is imperative for ethical research conduct with this population.

Smaller focus groups may be optimal for data collection among this population [8]. Data from the present analysis suggest that virtual synchronous groups among BSMM should be limited to 5 individuals to maximize participant comfort and privacy. Although some suggest that online focus groups should be between 8 and 12 participants [11,20], limiting group size provides an opportunity for rapport building between the focus group facilitator and participants as well as among participants. Limiting the size of the group also reduces the likelihood of compromising participants’ locations and lessens the risk of privacy breaches. Procedures to maintain participant safety during virtual synchronous focus groups should be described prior to study participation, and the facilitator should be prepared to remove participants who are unable to maintain privacy standards during online discussions.

Having a culturally congruent and culturally competent research team could be an integral part of participant sharing and satisfaction across focus groups [8]. Other studies have found that having culturally congruent research teams reduces research mistrust and fosters optimal participation among BSMM [8,10,33]. Although this study did not measure this directly, having culturally congruent teams could also reduce social desirability bias among BSMM [8,10,34]. Optimizing the utility of cultural congruence includes several factors, including an aesthetic component, personal disclosures, and providing extensive details related to the purpose and importance of the research and participation. Specifically, research teams can “look like” participants and share demographic characteristics, yet maintain professionalism [8,10]. Team members can also share personal information regarding their relationships to the community (eg, being a BSMM or not, living in similar communities or not), and passionately explain why their participation in the research study is important. These activities could build rapport and trust as well as reinforce the importance of maintaining safety standards. Still, research teams should be explicitly trained to optimize virtual synchronous focus group participation among BSMM regardless of demographic characteristics by explaining all study procedures, building trust, and establishing privacy procedures.

Incentivizing research participation is also important for this population. As medical and research mistrust persists for BSMM, immediately incentivizing participants after their study participation is crucial to limiting mistrust and skepticism despite the presence of a culturally congruent team. Due to COVID-19, incentive distribution was more delayed than that in usual in-person activities that permit the immediate disbursement of cash or gift cards. Ultimately, administrative regulations on virtual incentives were updated, and we were able to promptly disburse claim codes electronically upon completion of research participation.

Limitations should be acknowledged. The parent studies included convenience samples of BSMM who were recruited in part through snowball sampling, which limits the application of current methods on a more diverse sample of BSMM. We also conducted this study among HIV-negative BSMM; it is unclear whether these procedures would be equally acceptable among BSMM who are living with HIV. Additionally, this study included relatively small samples, and we were not able to quantify potential social desirability bias in responses.

However, few studies have detailed the important concepts in conducting virtual synchronous focus groups in HIV prevention research and among BSMM. The present considerations to maximize virtual synchronous focus group participation in HIV prevention research among BSMM align with existing recommendations for approaches in community-based research and cultural competency in clinical and research settings [8,24,35-37]. Future research should quantify the relative participation rates of virtual versus in-person focus groups and continue to explore preferences for ethical research study conduct in HIV prevention research among BSMM. Future research should also test the relative impact of larger versus smaller group sizes on group dynamics and participant responses. It is important that researchers take careful consideration of research conduct with this population and remind participants that they are a part of the process of reducing HIV and promoting community public health.

## Abbreviations

BSMM: Black sexual minority men
IRB: institutional review board
PI: principal investigator
PrEP: pre-exposure prophylaxis
